# Bacterial peptidoglycan primes the immune system leading to increased sickness in response to lipopolysaccharide

**DOI:** 10.1186/2050-6511-13-S1-A71

**Published:** 2012-09-17

**Authors:** Aitak Farzi, Evelin Painsipp, Peter Holzer

**Affiliations:** 1 Institute of Experimental and Clinical Pharmacology, Medical University of Graz, 8010 Graz, Austria

## Background

While the effects of the bacterial component and Toll-like receptor-4 (TLR4) agonist, lipopolysaccharide (LPS), on affective behaviour is well described, there is a lack of data concerning the effects of peptidoglycans on behaviour. Amongst others, peptidoglycans activate the intracellular receptors nucleotide-binding oligomerization domain 1 (NOD1) and NOD2. Here, the effects of the NOD1 activator FK565 and NOD2 activator muramyl dipeptide (MDP) were investigated with respect to parameters of immune activation and behaviour.

## Methods

Male C57BL/6N mice received an intraperitoneal injection of FK565 (0.003 mg/kg), MDP (3 mg/kg) or sterile saline (0.9% NaCl) and an additional injection of LPS (0.83 mg/kg) or sterile saline 4 hours after the first injection. Body weight and rectal temperature were monitored throughout the study. Exploratory and anxiety-like behaviour was evaluated with the open field test (OFT) 1 day and with the step-down test 2 days after treatment. Spleen weight, an index of immune activation, was measured on the third day after sacrifice of mice.

## Results

While none of the single treatments induced changes of body temperature, combined treatment with FK565+LPS and MDP+LPS caused a decrease of body temperature 4.5 hours post-treatment. A loss of body weight could be observed in the LPS, FK565+LPS and MDP+LPS-treated groups on day 1 and 2, while FK565 and MDP alone had no effect on body weight. On the third day post-treatment, the weight loss was gone in the LPS treated group, but was still evident in the groups receiving the double treatments. In the OFT, only treatment with FK565+LPS or MDP+LPS decreased travelling distance and visits to the central area. Likewise, in the step-down test only the double-treated mice presented an increased latency. LPS alone and in combination with FK565 or MDP increased spleen weight while FK565 and MDP alone were without effect.

## Conclusions

The present results reveal that administration of a NOD1 or NOD2 activator alone fails to induce any systemic signs of immune activation, sickness (weight loss) and behavioural disturbance. In contrast, mice primed with either FK565 or MDP display increased anxiogenic and immune reactions to LPS. These findings indicate that NOD and TLR-4 agonists synergize *in vivo* in causing immune activation and sickness behaviour.

